# Culture media affects accuracy of prediction of metallo-β-lactamases mediated resistance to imipenem

**DOI:** 10.1371/journal.pone.0341347

**Published:** 2026-01-28

**Authors:** Kexuan Chen, Sarah Miller, Kristine Goy, Tina Lam, Marlène Maeusli, Rosemary She, Brad Spellberg, Brian Luna

**Affiliations:** 1 Department of Immunology and Immune Therapeutics, Keck School of Medicine at USC, Los Angeles, California, United States of America; 2 Department of Pathology, City of Hope, Duarte, California, United States of America; 3 Los Angeles General Medical Center, Los Angeles, California, United States of America; Yamagata University Faculty of Medicine: Yamagata Daigaku Igakubu Daigakuin Igakukei Kenkyuka, JAPAN

## Abstract

Metallo-β-lactamases (MBLs) hydrolyze the beta-lactam ring in beta-lactam antibiotics, rendering them ineffective. Infections caused by MBL-harboring bacteria result in higher mortality, more costs and prolonged hospital stays due to the limited treatment options. Recently, antimicrobial susceptibility testing using RPMI-1640 has been found to have more accuracy in predicting *in vivo* efficacy due to its zinc deficiency. We sought to expand the previous studies of accessing *in vivo* efficacy using zinc-limited media versus conventional media to more antimicrobial agents and MBL-producing strains. The susceptibility of isolates was determined by performing minimum inhibitory concentration (MIC) assays using traditional cation-adjusted Mueller Hinton broth (CAMHB) or zinc-limited media. *In vivo* outcomes were evaluated using the *Galleria mellonella* infection model and neutropenic mouse thigh infection model. The MICs of MBL-harboring strains decreased in zinc-limited media compared to in nutrient-rich media, suggesting susceptibility of a subset of resistant strains when tested in zinc-limited media. Notably, we observed statistically significant MICs decreasing against imipenem, which demonstrated the best efficacy among the six tested antibiotics. Additionally, the outcomes of *in vivo* tests in both the *G. mellonella* model and the mouse model were better predicted with *in vitro* MIC assays performing in zinc-limited media. The use of zinc-limited media may lead to increased accuracy of the prediction of *in vivo* efficacy of beta-lactams against MBL-harboring bacteria.

## Introduction

Antibiotic-resistant bacteria impose a significant burden on healthcare. In 2017, the U.S. Centers for Disease Control and Prevention (CDC) estimated there were 2.8 million infections and more than 35,000 deaths due to infections caused by antibiotic-resistant bacteria in the U.S. [1]. These estimates are nearly double the previous estimates that were published in 2013 [1,2]. In 2019, global estimates were 4·95 million deaths associated with bacterial antimicrobial resistance [3].

Metallo-β-lactamases (MBLs) are a set of enzymes produced by some bacteria that can hydrolyze a broad range of β-lactam agents, including carbapenems. Carbapenems are potent and effective antibiotics used clinically for treatment of severe bacterial infections. Carbapenem resistance, especially in Gram-negative bacteria, derived from MBLs is a global healthcare problem and there are limited therapeutic options [4].

The hydrolyzation function of MBLs requires zinc cations [5]. Small molecule chelators, such as EDTA, have been shown to inhibit MBL activity; however, there is no Food and Drug Administration (FDA)approved inhibitor with activity against MBLs [6]. Recent reports have suggested that the supraphysiological concentration of cations in standard bacterial culture media results in discordance between the lack of *in vitro* activity of beta-lactams against MBL-harboring pathogens and the surprising *in vivo* efficacy of those same antibiotics [7–11]. These data suggest that standard *in vitro* antibiotic susceptibility testing methods overestimate the resistance contributions of the MBL enzyme to *in vivo* outcomes.

We recently found the mammalian cell culture medium RPMI-1640, which is relatively nutrient-depleted compared to traditional cation-adjusted Mueller Hinton broth (CAMHB), better predicted *in vivo* efficacy of rifabutin, azithromycin, and transferrin against carbapenem-resistant Gram-negative pathogens [12–18]. Similarly, other labs have also independently found that modified antimicrobial susceptibility testing conditions have better predicted *in vivo* outcomes for some specific drug/pathogen combinations [19–22]. In this study, we expand on prior results to evaluate the accuracy of RPMI-1640 versus CAMHB medium at predicting *in vivo* efficacy of 6 β-lactam antibiotics: ertapenem (ETC), imipenem (IPM), meropenem (MEM), piperacillin and tazobactam (TZP), ceftazidime (CAZ), and ceftriaxone (CRO) against 56 MBL-harboring clinical isolates that include *Klebsiella pneumoniae*, *Acinetobacter baumannii*, *Escherichia coli*, and *Pseudomonas aeruginosa*.

## Materials and methods

### Ethics statement

All animal work was conducted following approval by the Institutional Animal Care and Use Committee at the University of Southern California, in compliance with the recommendations in the Guide for the Care and Use of Laboratory Animals of the National Institutes of Health. Infected mice develop weight loss, ruffled fur, poor appetite, decreased ambulation, huddling behavior, and low body temperature. Mice were monitored at least twice daily for seven days. Mice that displayed huddling behavior and are poorly mobile were weighed once daily. Weight loss of greater than 15% pre-infection body weight triggered euthanasia via CO2 chamber and secondary cervical dislocation. Soft bedding and other enrichment devices were provided as recommended by the veterinary staff. Nutritional supplements such as hydrogel packs were provided as needed.

### Strain selection and preparation

Bacterial isolates were obtained from the CDC Antibiotic Resistance Isolate Bank. Bacteria are listed in **S1 Table**. Bacteria from frozen glycerol stocks were subcultured on tryptic soy agar (TSA) plates and incubated at 35 ± 2˚C in ambient air to obtain single colonies. For minimum inhibitory concentration (MIC) assays, fresh (18–24 hour), single colonies were selected and adjusted to 0.5 Macfarland solution.

### Antibiotics

Pharmaceutical grade ertapenem (ETC; NDC #72266-159-10; FOSUN PHARMA), imipenem and cilastatin (IPM; NDC #63323-349-25; FRESENIUS KABI), meropenem (MEM; NDC #0409-1390-51; Hospira), piperacillin and tazobactam (TZP; NDC #68001-506-30; BluePoint), ceftriaxone (CRO; NDC #0409-7332-20; novaplus), and ceftazidime (CAZ; NDC #0409-5084-13; Hospira) were stored at room temperature. Antibiotic agents were dissolved in water to make stock solutions. The breakpoints for each antibiotic used to interpret the MIC results are defined as follows: for meropenem (S/I/R): *A. baumannii*/*P. aeruginosa*: ≤ 2, 4, ≥ 8; for ertapenem (S/I/R): *A. baumannii*/*P. aeruginosa*: intrinsically resistant; for reference purposes, Enterobacterales breakpoints are shown; for imipenem (S/I/R): *A. baumannii*/*P. aeruginosa*: ≤ 2, 4, ≥ 8; for piperacillin-tazobactam (S/I/R): *A. baumannii*: ≤ 16, 32–64, ≥ 128; *P. aeruginosa*: ≤ 16, 32, ≥ 64; for CRO (S/I/R): *A. baumannii*: ≤ 8, 16–32, ≥ 64; *P. aeruginosa*: intrinsically resistant; for reference purposes, Enterobacterales breakpoints are shown; for ceftazidime (S/I/R): *A. baumannii*/*P. aeruginosa*: ≤ 8, 16, ≥ 32 [23].

### MIC assay

The broth microdilution method was used to determine MICs [12,24]. The medium used for MIC testing was either CAMHB (212322; BD Biosciences) or the zinc-limited medium CAMHB + 30 mg/L EDTA and RPMI-1640 (11875119; Thermo Fisher). Briefly, using the colony suspension method, after 18–24 hour growth on non-selective agar, 3–5 isolated colonies were suspended in phosphate-buffered saline (PBS) and adjusted to achieve a 0.5 McFarland solution. The organism suspension was added to wells of a 96-well plate containing serial drug dilutions in culture media (CAMHB, CAMHB + 30 mg/L EDTA, or RPMI-1640) to a final concentration of 5 x 10^5^ CFU/mL. Antibiotics covered a range of 2-fold serial dilutions from 0.25 to 128 mg/L. Each strain was tested in duplicate. MICs were recorded after plate incubation at 35 ± 2°C for 20–24 hr in ambient air [24].

In the MIC assays using IPM and AVI combination, the concentration of avibactam was held constant at 4 mg/L and combined with a range of 2-fold serial dilutions of imipenem from 0.25 to 128 mg/L.

### *Galleria mellonella* infection model

The larvae of *Galleria mellonella* were kept at room temperature in the dark until used for experiments. Healthy larvae with yellow bodies weighing 180–330 mg were randomly grouped by tens. Larvae were immobilized by incubating at 4°C for approximately 40 minutes prior to injection. The larvae were surface sterilized by being gently wiped using a 70% ethanol-soaked KimWipe and then stored in a clean Petri dish on ice. For injection, each larva was restrained in a folded sponge as previously described [15,25]. An antibiotic solution or bacterial inoculum was filled in a 1 mL syringe with a 30G needle, and 10 µL was injected into each larva through its most posterior proleg(s) using a syringe pump (New Era Pump Systems, Inc.). After all the injections, the larvae were incubated at 35 ± 2°C, and survival was monitored for 4 days post-infection.

### Neutropenic mouse thigh infection model

Healthy 8–9 weeks male C3HeB/FeJ (Jackson Laboratory, stock no. 000658) mice were driven neutropenic by receiving 150 mg/kg and 100 mg/kg cyclophosphamide via intraperitoneal (IP) injection 4 days and 1 day before the infection. Each thigh of the mouse was inoculated with 0.1 mL 1E8 CFU/mL bacterial suspension in PBS. After infection, one mouse would be euthanized immediately, and another at 2 hours post-infection. The thighs were then collected and homogenized in 2 mL PBS for the drop plating, and the CFU per thigh was determined after 24 hours of incubation at 37°C. The remaining mice were divided into two groups (n = 3) receiving treatment subcutaneously with either 250 μL 25 mg/kg imipenem or an equivalent volume of PBS at 2, 6, 10 and 22 hours post-infection [26]. All the mice were euthanized at 26 hours for thigh collection. All animal procedures were conducted with approval from the Institutional Animal Use and Care Committee (IACUC) at the University of Southern California.

### Statistics

Statistics were performed using R. Pairwise comparisons were evaluated using the Mann-Whitney U test. Time to death was compared using the Log Rank test. P values < 0.05 were considered significant.

## Results

**Fig 1:**
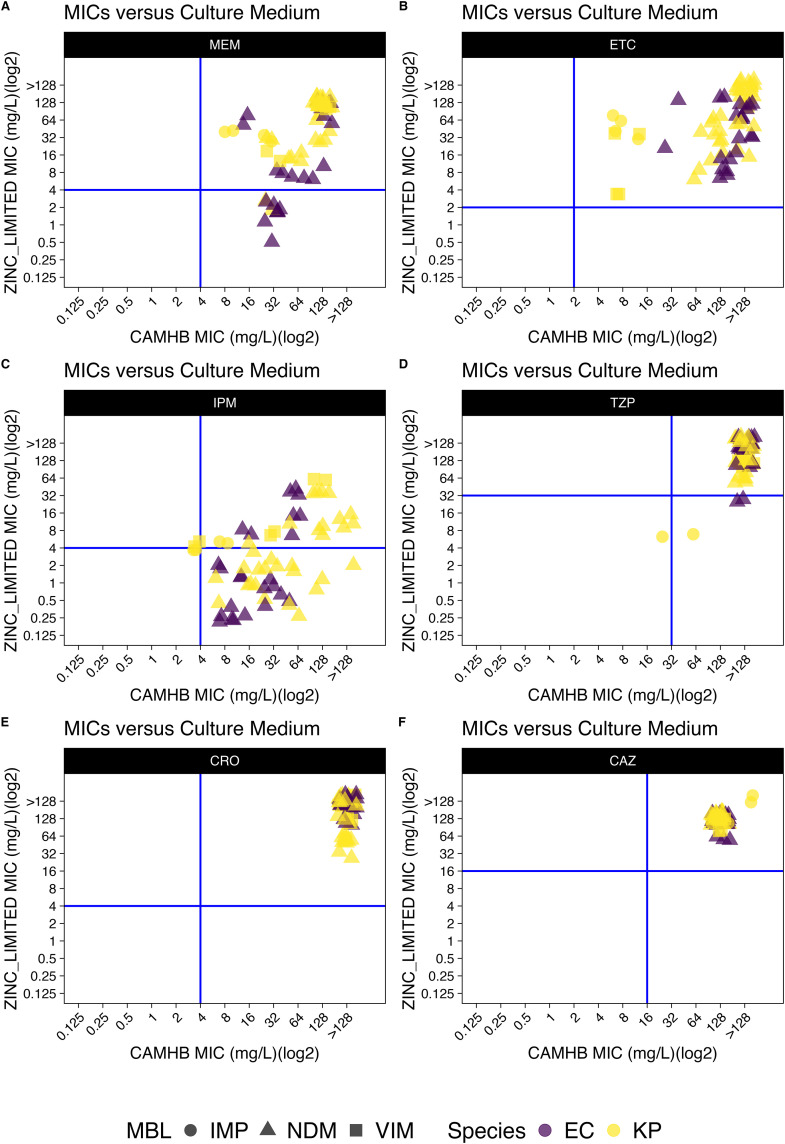
MIC of *E. coli* and *K. pneumoniae* in CAMHB medium versus MIC in zinc-limited media. MICs for 6 antibiotics (MEM, ETC, IPM, TZP, CRO, CAZ, A to F) were determined using either CAMHB or RPMI-1640 medium against 33 metallo-β-lactamase-producing clinical isolates: *K. pneumoniae* (n = 21), and *E. col*i (n = 12). **(A)** MIC distributions for MEM. **(B)** MIC distributions for ETC. **(C)** MIC distributions for IPM. **(D)** MIC distributions for TZP. **(E)** MIC distributions for CRO. **(F)** MIC distributions for CAZ. Breakpoints (μg/mL) are defined as follows: For MEM (S/I/R): *E. coli/K. pneumoniae*: ≤ 1, 2, ≥ 4; For ETC (S/I/R): *E. coli/K. pneumoniae*: ≤ 0.5, 1, ≥ 2; For IPM (S/I/R): *E. coli/K. pneumoniae*: ≤ 1, 2, ≥ 4; For TZP (S/I/R): *E. coli/K. pneumoniae*: ≤ 8, 16, ≥ 32; For CRO (S/I/R): *E. coli/K. pneumoniae*: ≤ 1, 2, ≥ 4; For CAZ (S/I/R): *E. coli/K. pneumoniae*: ≤ 4, 8, ≥ 16. The blue solid lines indicate the breakpoints. Everything to the right of the vertical line is predicted to be resistant in CAMHB and everything upon the horizontal line is predicted to be resistant in zinc-limited media. Right lower quadrant represents isolates resistant in CAMHB but susceptible in zinc-limited media. Abbreviations: imipenemase (IMP), New Delhi metallo-beta-lactamase (NDM), Verona integron-encoded metallo-beta-lactamase (VIM), metallo-β-lactamases (MBL).

**Fig 2:**
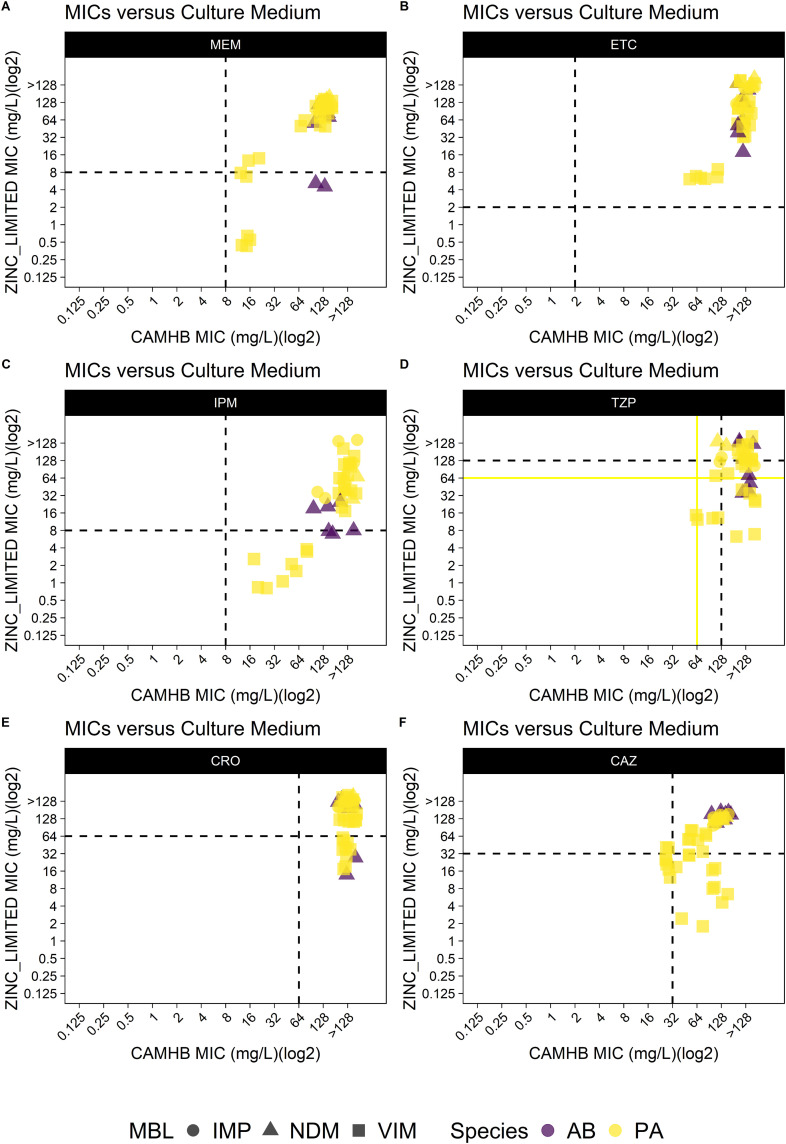
MIC of *A. baumannii* and *P. aeruginosa* in CAMHB medium versus MIC in zinc-limited media. MICs for 6 antibiotics (MEM, ETC, IPM, TZP, CRO, CAZ, A to F) were determined using either CAMHB or CAMHB_EDTA medium against 23 metallo-β-lactamase-producing clinical isolates: *A. baumannii* (n = 4), and *P. aeruginosa* (n = 19). **(A)** MIC distributions for MEM. **(B)** MIC distributions for ETC. **(C)** MIC distributions for IPM. **(D)** MIC distributions for TZP. **(E)** MIC distributions for CRO. **(F)** MIC distributions for CAZ. Breakpoints (μg/mL) are defined as follows: For MEM (S/I/R): *A. baumannii*/*P. aeruginosa*: ≤ 2, 4, ≥ 8; For ETC (S/I/R): *A. baumannii*/*P. aeruginosa*: intrinsically resistant; for reference purposes, Enterobacterales breakpoints are shown; For IPM (S/I/R): *A. baumannii*/*P. aeruginosa*: ≤ 2, 4, ≥ 8; For TZP (S/I/R): *A. baumannii*: ≤ 16, 32-64, ≥ 128; *P. aeruginosa*: ≤ 16, 32, ≥ 64; For CRO (S/I/R): *A. baumannii*: ≤ 8, 16-32, ≥ 64; *P. aeruginosa*: intrinsically resistant; for reference purposes, *A. baumannii* breakpoints are shown; For CAZ (S/I/R): *A. baumannii*/*P. aeruginosa*: ≤ 8, 16, ≥ 32. The black dashed lines indicate the breakpoints for *A. baumannii*. The yellow solid lines indicate the breakpoints for *P. aeruginosa*. Everything to the right of the vertical line is predicted to be resistant in CAMHB and everything upon the horizontal line is predicted to be resistant in zinc-limited media. Right lower quadrant represents isolates resistant in CAMHB but susceptible in zinc-limited media. Abbreviations: imipenemase (IMP), New Delhi metallo-beta-lactamase (NDM), Verona integron-encoded metallo-beta-lactamase (VIM), metallo-β-lactamases (MBL).

**Fig 3:**
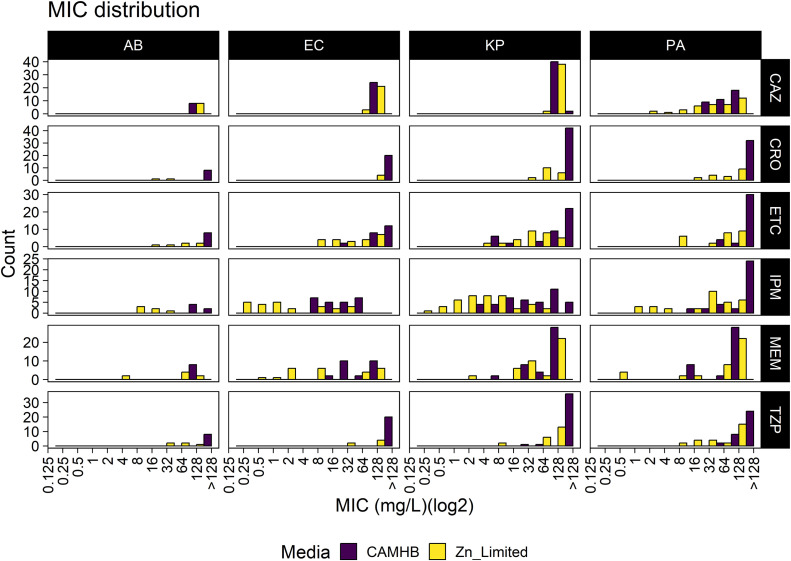
MIC distribution histogram. MICs for 6 antibiotics (MEM, ETC, IPM, TZP, CRO, CAZ) were determined using either CAMHB or zinc-limited media against 56 metallo-β-lactamase-producing clinical isolates: *A. baumannii* (n = 4), *E. coli* (n = 12), *K. pneumoniae* (n = 21), and *P. aeruginosa* (n = 19). Abbreviations: meropenem (MEM), ertapenem (ETC), imipenem and cilastatin (IPM), piperacillin and tazobactam (TZP), ceftriaxone (CRO), ceftazidime (CAZ), *A.* *baumannii* (AB), *E. coli* (EC), *K. pneumoniae* (KP), *P. aeruginosa* (PA).

**Fig 4:**
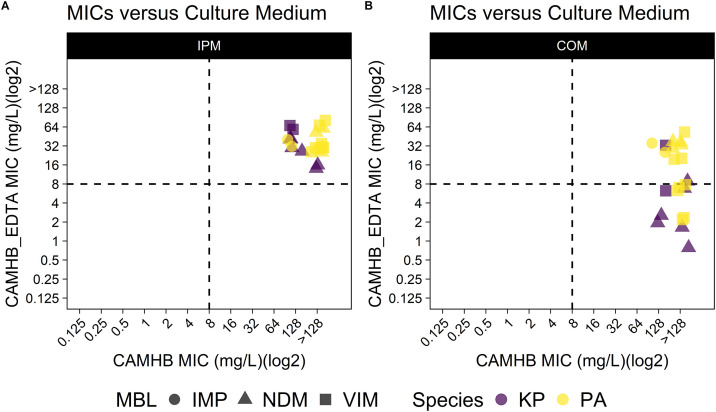
IPM in combination with AVI. MICs were determined against 11 *K. pneumoniae*, and *P. aeruginosa* strains in CAMHB and CAMHB_EDTA media **(A to B)**. **(A)**. The therapeutic agent was IPM. **(B)**. The therapeutic agent was a combination of IPM and AVI. The breakpoint was 8 mg/L. Statistical comparisons were made between the MICs values against two different drug groups when tested in zinc-limited media (Mann-Whitney; p < 0.001).

We determined the MICs for meropenem, ertapenem, imipenem, piperacillin and tazobactam, ceftriaxone, and ceftazidime against a panel of 56 metallo-β-lactamase-producing clinical isolates, including *Klebsiella pneumoniae* (n = 21), *Acinetobacter baumannii* (n = 4), *Escherichia coli* (n = 12), and *Pseudomonas aeruginosa* (n = 19) (Figs 1–4). CAMHB supplemented with 30 mg/L EDTA, or RPMI-1640 were used as the culture medium via the broth microdilution method per the Clinical and Laboratory Standards Institute (CLSI). EDTA was used as a metal ion chelator as previously described [7]. Because many *P. aeruginosa* clinical isolates were not able to grow in RPMI-1640, we used CAMHB supplemented with EDTA instead.

The susceptibility breakpoints of meropenem, ertapenem, imipenem, piperacillin and tazobactam, ceftriaxone, and ceftazidime have been determined by CLSI for *E. coli*, *K. pneumoniae*, *A. baumannii* and *P. aeruginosa*. However, for ceftriaxone and ertapenem, due to the intrinsic resistance, there is no ertapenem susceptibility breakpoint for *P. aeruginosa* and *A. baumannii*. Therefore, *E. coli* breakpoints were used for reference purposes. Similarly, ceftriaxone and *P. aeruginosa* referenced established *A. baumannii* breakpoints. In CAMHB, only one strain was considered ceftazidime susceptible. All the rest of the isolates were considered resistant to all 6 drugs based on MICs in CAMHB. However, of the 56 isolates, 15 (26.8%) isolates were considered imipenem susceptible, 1 (1.8%) were considered meropenem susceptible, 2 (3.6%) were considered TZP susceptible, and 3 (5.4%) were considered ceftazidime susceptible based on MICs tested in zinc-limited media. No isolates were considered susceptible to either ceftriaxone or ertapenem in either CAMHB and zinc-limited media **(**Table 1**)**.

**Table 1:** Summary of MICs. “S” = Susceptible, “I” = Intermediate, “R” = Resistant.

	MEM		ETC		IPM		TZP		CRO		CAZ	
	CAMHB	Low-Zinc	CAMHB	Low-Zinc	CAMHB	Low-Zinc	CAMHB	Low-Zinc	CAMHB	Low-Zinc	CAMHB	Low-Zinc
***A. baumanni* (n = 4)**												
**S (%)**	0	0	0	0	0	0	0	0	0	0	0	0
**I (%)**	0	1 (25%)	0	0	0	1 (25%)	0	2 (50%)	0	1 (25%)	0	0
**R (%)**	4 (100%)	3 (75%)	4 (100%)	4 (100%)	4 (100%)	3 (75%)	4 (100%)	2 (50%)	4 (100%)	3 (75%)	4 (100%)	4 (100%)
***E. coli (n = 12)***												
**S (%)**	0	1 (8.3%)	0	0	0	7 (58.3%)	0	0	0	0	0	0
**I (%)**	0	3 (25.0%)	0	0	0	1 (8.3%)	0	0	0	0	0	0
**R (%)**	12 (100%)	8 (66.7%)	12 (100%)	12 (100%)	12 (100%)	4 (33.3%)	12 (100%)	12(100%)	12 (100%)	12 (100%)	12 (100%)	12 (100%)
***K. pneumoniae (n = 21)***												
**S (%)**	0	0	0	0	0	5 (42.9%)	0	1 (4.8%)	0	0	0	0
**I (%)**	0	1 (4.8%)	0	0	0	4 (19.0%)	0	0	0	0	0	0
**R (%)**	21 (100%0	20 (95.2%)	21 (100%)	21 (100%)	21 (100%)	12 (57.1%)	21 (100%)	20 (95.2%)	21 (100%)	12 (100%)	21 (100%)	21 (100%)
***P. aeruginosa (n = 19)***												
**S (%)**	0	2 (10.5%)	0	0	0	3 (15.8%)	0	3 (15.8%)	0	0	0	3 (15.8%)
**I (%)**	0	0	0	0	0	1 (5.3%)	0	2 (10.5%)	0	3 (15.8%)	1 (5.6%)	3 (15.8%)
**R (%)**	19 (100%0	17 (90.5%)	19 (100%)	19 (100%)	19 (100%)	15 (78.9%)	19 (100%)	14 (73.7%)	19 (100%)	16 (84.2%)	18 (94.4%)	13 (68.4%)
**Total (n = 56)**												
**S (%)**	0	3 (5.4%)	0	0	0	15 (26.8%)	0	4 (7.1%)	0	0	0	3 (5.4%)
**I (%)**	0	5 (8.9%)	0	0	0	7 (12.5%)	0	4 (7.1%)	0	3 (5.4%)	1 (1.8%)	3 (5.4%)
**R (%)**	56 (100%)	48 (85.7%)	56 (100%)	56 (100%)	56 (100%)	34 (60.7%)	56 (100%)	48 (85.8%)	56 (100%)	53 (94.6%)	55 (98.2%)	50 (89.3%)

Breakpoints (μg/mL) are defined as follows:

For MEM (S/I/R): *E. coli*/*K. pneumoniae*: ≤ 1, 2, ≥ 4; *A. baumannii*/*P. aeruginosa*: ≤ 2, 4, ≥ 8;

For ETC (S/I/R): *E. coli*/*K. pneumoniae*: ≤ 0.5, 1, ≥ 2; *A. baumannii*/*P. aeruginosa*: no CLSI breakpoints, *E. coli* breakpoints were used;

For IPM (S/I/R): *E. coli*/*K. pneumoniae*: ≤ 1, 2, ≥ 4; *A. baumannii*/*P. aeruginosa*: ≤ 2, 4, ≥ 8;

For TZP (S/I/R): *E. coli*/*K. pneumoniae*: ≤ 8, 16, ≥ 32; *A. baumannii*: ≤ 16, 32–64, ≥ 128; *P. aeruginosa*: ≤ 16, 32, ≥ 64;

For CRO (S/I/R): *E. coli*/*K. pneumoniae*: ≤ 1, 2, ≥ 4; *A. baumannii*: ≤ 8, 16–32, ≥ 64; *P. aeruginosa*: no CLSI breakpoints, *A. baumannii* breakpoints were used;

For CAZ (S/I/R): *E. coli*/*K. pneumoniae*: ≤ 4, 8, ≥ 16; *A. baumannii*/*P. aeruginosa*: ≤ 8, 16, ≥ 32.

We observed that 22 of 56 (39.3%) isolates that were resistant to imipenem in CAMHB were found to be intermediate or susceptible in RPMI-1640 or CAMHB supplemented with EDTA **(**Fig 1**)**, and there was a significant difference in the distribution of the imipenem MICs (Mann-Whitney; KP: p < 0.01; AB: p < 0.01; EC: p < 0.01; PA: p < 0.01).

The blue solid lines indicate the breakpoints. Everything to the right of the vertical line is predicted to be resistant in CAMHB and everything upon the horizontal line is predicted to be resistant in zinc-limited media. Right lower quadrant represents isolates resistant in CAMHB but susceptible in zinc-limited media.

While we did not observe qualitative growth differences when bacteria were cultured in CAMHB or CAMHB supplemented with EDTA, it remains possible that EDTA may promote membrane permeabilization. To address this concern, we performed the modified carbapenem inactivation method (mCIM) and the EDTA-modified carbapenem inactivation method (eCIM). For these tests, a standard meropenem disk used for disk diffusion assays is first incubated with the clinical isolate in normal CAMHB or CAMHB supplemented with EDTA. After incubation with the clinical isolate, the disk is then transferred to an agar plate for a disk diffusion assay against a MEM-susceptible reporter strain. If the clinical isolate expresses carbapenemase activity in either condition, then the amount of MEM in the disk is reduced and the reporter stain would show improved growth in the disk diffusion assay. Of the isolates that had a positive result for the mCIM test, 8/9 (88.9%) isolates also tested positive for the eCIM test, an outcome that is consistent with having a functional MBL (Table 2).

**Table 2:** Carbapenem inactivation method (mCIM) and EDTA-modified carbapenem inactivation method (eCIM) results.

Species	Strain	mCIM Result	eCIM Result
*Pseudomonas aeruginosa*	0111	Negative	–
*Pseudomonas aeruginosa*	0230	Negative	–
*Pseudomonas aeruginosa*	0240	Negative	–
*Pseudomonas aeruginosa*	0241	Positive	Positive
*Pseudomonas aeruginosa*	0242	Positive	Positive
*Pseudomonas aeruginosa*	0243	Positive	Positive
*Pseudomonas aeruginosa*	0245	Positive	Positive
*Pseudomonas aeruginosa*	0246	Positive	Positive
*Pseudomonas aeruginosa*	0248	Positive	Positive
*Pseudomonas aeruginosa*	0250	Positive	Positive
*Pseudomonas aeruginosa*	0254	Negative	–
*Pseudomonas aeruginosa*	0255	Negative	–
*Pseudomonas aeruginosa*	0100	Negative	–
*Pseudomonas aeruginosa*	0103	Positive	Positive
*Pseudomonas aeruginosa*	0108	Negative	–
*Pseudomonas aeruginosa*	0110	Negative	–
*Pseudomonas aeruginosa*	0054	Negative	–
*Pseudomonas aeruginosa*	0092	Positive	Negative
*Pseudomonas aeruginosa*	0249	Negative	–
*Klebsiella pneumoniae*	ATCC BAA 1705	Positive	Negative
*Klebsiella pneumoniae*	ATCC BAA 1706	Negative	–
*Klebsiella pneumoniae*	ATCC BAA 2146	Positive	Positive

The mCIM and eCIM assays were performed in triplicate for each isolate, and the results were consistent across independent triplications. *Klebsiella pneumoniae* ATCC BAA-1705, 1706, 2146 were included as quality controls, and their results were consistent with expected phenotypes. For the mCIM test, a zone diameter of 6–15 mm is interpreted as a “positive” result for carbapenamase activity. For the eCIM test, the result is only interpretable if the mCIM test result is first “positive”. A “positive” result for the eCIM test is defined as a >= 5 mm increase in the zone diameter for the eCIM condition as compared to the mCIM condition.

The beta-lactamase inhibitor avibactam can inhibit serine beta-lactamases but not MBLs. To determine if additional isolates would become susceptible to imipenem in the presence of avibactam in the zinc-limited environment, we tested 11 *K. pneumoniae* and *P. aeruginosa* clinical isolates that were all resistant to imipenem in both CAMHB and CAMHB supplemented with EDTA. There was a significant difference between the MICs values against imipenem or AVI + IPM when tested in CAMHB supplemented with EDTA medium (Fig 4) (Mann-Whitney;KP: p = 0.00314; PA: p = 0.00452).

To further investigate if CAMHB or zinc-limited media can better predict *in vivo* outcomes, we used the *G. mellonella* infection and treatment model. We chose imipenem as a therapeutic agent because it showed the best *in vitro* efficacy in the previously described MIC experiments. Six of *K. pneumoniae* strains were selected and divided into two groups (Table 3). *K. pneumoniae* 49, 158, 560 were resistant to imipenem in CAMHB while susceptible to imipenem in zinc-limited media. *K. pneumoniae* 46, 68, 153 were resistant in both CAMHB and zinc-limited media. The inoculum for infection was determined by titration and the lowest inoculum that reliably resulted in a lethal infection was used for treatment experiments.

**Table 3:** MIC values of selected strains for *in vivo* testing. KP49, KP158 and KP560 were IPM resistant in CAMHB while susceptible in RPMI-1640; KP49, KP68 and KP 153 were IPM resistant in both media.

Strain	Sequence Typing	CAMHB, IPM MIC (mg/L)	RPMI-1640, IPM MIC (mg/L)
**KP49**	ST11	128	1
**KP158**	NA	16	1
**KP560**	14	64	0.5
**KP46**	ST147	128	64
**KP68**	ST14	128	32
**KP153**	NA	128	32

**Fig 5:**
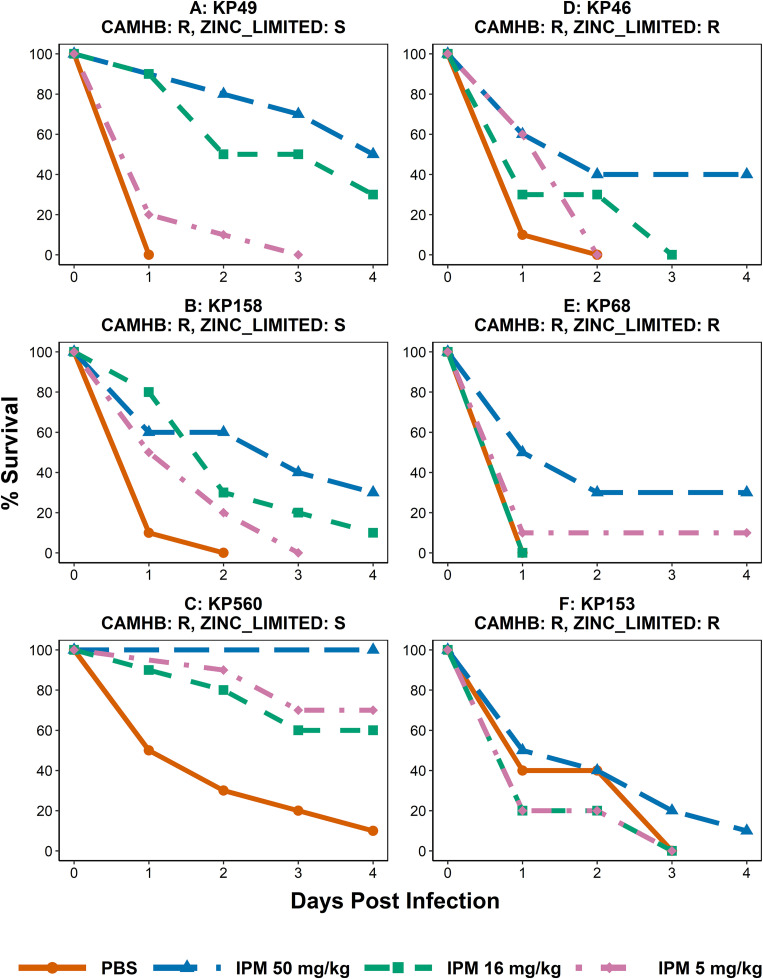
IPM efficacy in *Galleria mellonella* infection model. *Galleria mellonella* larvae (n = 10 per group) were infected and treated with IPM and monitored for survival **(A to F)**. KP49, KP158 and KP560 were resistant in CAMHB while susceptible in RPMI-1640 **(A to C)**. KP46, KP68 and KP153 were resistant in both CAMHB and RPMI-1640 **(D to F)**. The larvae were infected with lethal inoculum of KP49 (2.9E8 CFU/larva), KP158 (3.5E8 CFU/larva), KP560 (2E7 CFU/larva), KP46 (1.7E8 CFU/larva), KP68 (2.85E8 CFU/larva), KP153 (2.8E7 CFU/larva) before being treated with PBS or IPM. Statistical comparisons between PBS treatment and each IPM treatment group were made using the log-rank test to compare survival.

*G. mellonella* larvae (n = 10) in the treatment group were challenged with a lethal inoculum and treated with either 5, 16, or 50 mg/kg imipenem. The control group were infected with a lethal inoculum but treated with PBS instead. At the dose 50 mg/kg, imipenem was able to rescue larvae infected with the first group of strains, which were resistant in CAMHB while susceptible in zinc-limited media (Log Rank test; p value: KP49 < 0.001; KP158 = 0.035; KP560 < 0.001, respectively). At the dose 16 mg/kg, imipenem was able to rescue larvae infected with KP49 (p=<0.001) and KP158 (p = 0.019). At the dose 5 mg/kg, imipenem was able to rescue larvae infected with KP560 (p = 0.022) (**Fig 5**).

For the group of strains that tested resistant to imipenem in both media conditions, there was no statistically significant difference in survival comparing the PBS control group to any of the imipenem treatment groups. These survival data were consistent with the results of previous *in vitro* experiments performed with zinc-low media while in contrast to results in CAMHB.

**Fig 6:**
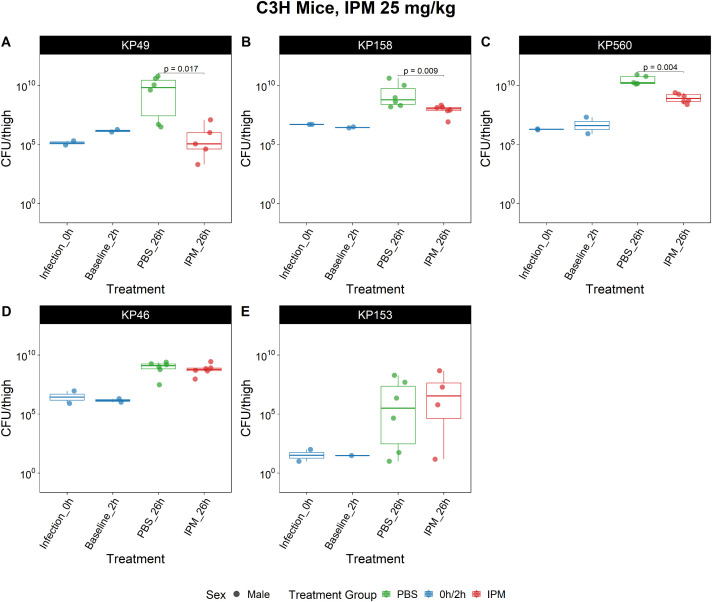
IPM efficacy in neutropenic mouse thigh infection model. Neutropenic mice were challenged with different *K. pneumoniae* clinical isolates. Each thigh of the mouse was inoculated with 0.1 mL 1E8 CFU/mL bacterial suspension in PBS; except for KP153, which received 1E3 CFU/mL inoculum. After infection, one mouse would be euthanized immediately, and another at 2 hours post-infection. The remaining mice were administered treatment subcutaneously with either 25 mg/kg imipenem or PBS at 2, 6, 10 and 22 hours post-infection. All the mice were euthanized at 26 hours for thigh collection. There were significant differences between PBS groups and treatment groups in susceptible strains (A-C; KP49: p = 0.017; KP158: p = 0.009; KP560: p = 0.004, Mann-Whitney), while not observed in strains resistant to imipenem **(D-E)**.

Because imipenem pharmacokinetics are unknown in a *Galleria mellonella* infection model, we next evaluated imipenem efficacy in a neutropenic mouse thigh infection model that utilized previously validated dosing strategy [26]. Of the six strains tested in the *G. mellonella* model, five were also evaluated in the thigh model, except for the resistant strain *K. pneumoniae* 68. Each thigh was initially infected with 1E7 CFU, at 0 and 2 hours post-infection, the bacterial CFUs were counted ranging between 1E5 to 1E7 per thigh, which increased to 1E8 to 1E10 CFUs per thigh by 26 hours under the treatment with PBS. There was a significant reduction in bacterial burden in the mice receiving treatment with 0.25mL 25 mg/kg imipenem q4h (Mann-Whitney; p value: KP49 = 0.017; KP158 = 0.009; KP560 = 0.004), while this trend has not been observed in the mice group administering a placebo. (**Fig 6**) Importantly, imipenem effectively maintained bacterial CFU levels close to baseline throughout the treatment period. Specifically, the inoculum of KP153 was down to 1E3 due to high virulence, even the initial inoculum was very low, the highest CFUs per thigh has reached to a maximum of 1E8, which was an astonishing 5-log increase.

## Discussion

Carbapenem resistance remains a paramount concern for healthcare. A significant challenge is that there are no FDA-approved inhibitors that are effective against MBLs. Unlike other beta-lactamases, MBLs require metal ions, typically zinc, for their substrate binding and catalytic activity. However, unlike standard *in vitro* culture conditions in which zinc is highly abundant, it is well recognized that zinc is poorly bioavailable in the host environment during infection [27]. Thus, we hypothesized that standard susceptibility testing in enriched media might not accurately predict the efficacy of antibiotics targeted by MBLs when used to treat infections in the normally zinc-sequestered *in vivo* environment.

As shown in **Fig 1**, compared to nutrient rich media, the MIC values decreased when tested in zinc-limited media. Especially for imipenem, it was found that one third of screened strains were no longer resistant to imipenem when tested in zinc-limited media. We found that avibactam restored efficacy of imipenem to some, but not all *K. pneumoniae* strains tested. Avibactam will effectively inhibit serine-beta lactamases but not MBLs. Therefore, for the strains that were not susceptible in the presence of avibactam, there may be other non-beta-lactamase conferring resistance mechanisms such as porin mutations that still contribute to resistance.

When tested in zinc-limited media instead of CAMHB medium, the results of *in vitro* MIC assays showed more consistency with the *in vivo* outcomes using the *G. mellonella* model. However, as an invertebrate animal, *G. mellonella* lacks the complex immune system of humans, and therefore cannot adequately capture pharmacokinetics, the processes by which a drug is absorbed, distributed, metabolized, and excreted in the body. To identify and find dosing strategies that better match the pharmacokinetics in humans, we then utilized a murine neutropenic thigh infection model to further validate our assumptions. Our results indicated that our *in vitro* data also successfully predicted the *in vivo* outcomes in the murine model.

Clinically, imipenem is usually administered by continuous infusion every 6 or 8 hours to maximize the fT > MIC. To replicate this in mice, imipenem was administered every 4 hours based on a previously established human-simulated regimen which would result in approximately 24% fT > MIC, a value that is considered the minimum value necessary for a bacteriostatic effect [26,28]. However, given the 13-minute half-life of imipenem, it remains a significant challenge to administer the imipenem frequently enough to mice to fully replicate the pharmacokinetic profile observed in human patients. Nevertheless, the observed benefit of treatment as compared to the PBS control group is only predicted by the MICs determined in the low zinc media.

Compared to meropenem, which is more widely used in clinical practice, imipenem may have the potential to treat infections caused by MBL-harboring pathogens. Additional research is needed to better characterize MBL activity against different beta-lactam substrates in low zinc conditions.

One limitation of this study is that we were unable to identify if the specific type of MBL (NDM, VIM, or IMP) was more or less affected by the low zinc environment. Due to the heterogeneity of the genotypes of the unique clinical isolates, and the relatively few number of IMP and VIM isolates in our collection, we were unable to address the question.

Other reported studies have also shown that carbapenems remain effective against MBL harboring pathogens when antibiotic susceptibility testing is conducted with low-zinc media. Our data are consistent in that some, but not all, *P. aeruginosa*, *K. pneumoniae*, and *E. coli* clinical isolates become more susceptible to meropenem in low-zinc media [7,11]. Previous reports did not evaluate imipenem, but our study found imipenem to be superior to meropenem. However, due to the lack of the evaluation using of animal model for MEM, future *in vivo* work is required for the further validation.

The MIC shifts differed among β-lactam agents, suggesting the possibility of compound-specific interactions with zinc limited media. Although the underlying mechanisms were not explored in this study, it is important to recognize the variations, and future comparisons between multiple β-lactam agents and media conditions will be needed.

In summary, we find that standard *in vitro* susceptibility testing of beta-lactam antibiotics against bacterial isolates containing MBL enzymes may not accurately predict the resistance of the bacteria to these standard antibiotics, due to lack of available zinc co-factor in the *in vivo* setting. These results add to the growing body of studies that emphasize limitations of the accuracy of susceptibility testing in nutrient rich media for some antibiotic-pathogen combinations, and suggest that clinical studies of the effectiveness of beta-lactam drugs against MBL-containing bacteria may be warranted.

## Supporting information

S1 Table Clinical isolates used in this study. The clinical isolates and known beta-lactamases are summarized.
(XLSX)
